# Cortisol:brain-derived neurotrophic factor ratio associated with silent ischaemia in a black male cohort: the SA BPA study

**DOI:** 10.5830/CVJA-2016-065

**Published:** 2016

**Authors:** Christiaan E Schutte, Leoné Malan, Jacobus D Scheepers, Woudri Oosthuizen, Nicolaas T Malan, Marike Cockeran

**Affiliations:** Hypertension in Africa Research Team (HART ), North-West University, Potchefstroom Campus, South Africa; Hypertension in Africa Research Team (HART ), North-West University, Potchefstroom Campus, South Africa; Hypertension in Africa Research Team (HART ), North-West University, Potchefstroom Campus, South Africa; Hypertension in Africa Research Team (HART ), North-West University, Potchefstroom Campus, South Africa; Hypertension in Africa Research Team (HART ), North-West University, Potchefstroom Campus, South Africa; Statistical Consultation Services, North-West University, Potchefstroom Campus, South Africa

**Keywords:** cortisol, brain-derived neurotrophic factor (BDNF), cardiometabolic disease

## Abstract

**Aim:**

Emotional distress has been associated with cardiovascular disease (CVD) in Africans. Cortisol and brain-derived neurotrophic factor (BDNF), as markers of emotional distress, increase cardiometabolic risk. We therefore aimed to investigate associations between cardiometabolic risk markers and the cortisol-to-BDNF ratio (cortisol:BDNF).

**Methods:**

A cross-sectional study included a bi-ethnic gender cohort (n = 406) aged 44.7 ± 9.52 years. Ambulatory blood pressure (ABPM), ECG, fasting serum cortisol and BDNF levels and cardiometabolic risk markers were obtained.

**Results:**

Africans had increased incidence of hyperglycaemia and 24-hour silent ischaemic events, and elevated 24-hour blood pressure (BP) and cortisol:BDNF ratios compared to Caucasians. Forward stepwise linear regression analysis underscored a similar trend with associations between hyperglycaemia, 24-hour BP [Adj R2 0.21–0.29; β 0.23 (0.1–0.4); p = 0.01], silent ischaemia [Adj R2 0.22; β 0.40 (0.2–0.6); p < 0.01] and cortisol:BDNF levels in Africans, mostly in the men.

**Conclusion:**

Attenuated cortisol levels in this group may be indicative of emotional distress and if chronic, drive the cortisol:BDNF ratio to desensitise BDNF. Desensitised cortisol:BDNF may sustain cardiometabolic risk and induce neurodegeneration in African men via silent ischaemia. Compensatory increases in blood pressure to increase perfusion and maintain homeostasis may increase coronary artery disease risk.

## Aim

Cardiovascular disease (CVD) is a major concern throughout the world.^1^ There are various factors that contribute to the risk of CVD, such as alcohol abuse, obesity and urbanisation.^2^ A significant contributing risk factor for CVD, stroke and ischaemic heart disease is high blood pressure.3 Indeed, the South African National Health and Nutrition Examination Survey (SANHANES) found that systolic hypertensive rates ranged from 19.0 to 29.4% and diastolic hypertensive rates ranged from 8.3 to 19.4%. The prevalence of hypertension and psychological distress is escalating in South Africa, especially in urban communities.^3^-^5^

Prolonged exposure to a taxing emotionally stressful environment, such as an urban lifestyle, disrupts homeostasis, which can lead to chronic stress.^6^,^7^ A major pathway for regulating the stress response, the hypothalamic–pituitary–adrenal (HPA) axis, secretes corticotropin-releasing factor (CRF). CRF induces the release of adrenocorticotropic hormone (ACTH), where it stimulates the synthesis and secretion of glucocorticoids, with cortisol as the end product.^6^ Furthermore it has been observed that augmented HPA-axis responses towards challenges in normotensive individuals enhance the risk of developing hypertension.^8^ Dysregulation of the HPA axis may also be a result of unmitigated increases in cortisol where prolonged elevations ultimately result in the down-regulation of CRF, ACTH and subsequently cortisol.^9^

Mineralocorticoid-based hypertension is associated with an excess of extracellular fluid as a result of increased sodium and water retention.^7^ Resultant elevations in blood pressure will emerge with increased total peripheral resistance responses, which may burden the heart. This may contribute to silent ischaemic events because of a decrease in coronary blood supply.^3^

Brain-derived neurotrophic factor (BDNF), a protein complex and part of the neurotrophin family, is synthesised and secreted by the central nervous system and plays a major role in brain plasticity and survival of the developing neurons.^10^ The neurotrophic hypothesis of depression states that during times of stress, BDNF is down-regulated, especially in the limbic areas, influencing emotional responses.^7^ It is suggested that the downregulation is caused by corticosterone.^11^

The down-regulation may reduce neuroplasticity and ultimately lead to neurodegeneration. As the reduction by itself may not be enough to lead to destruction of hippocampal neurons, it may lead to an increased vulnerability to neuronal damage, especially during times of emotional distress.^11^,^12^ This suggests that attenuated levels of BDNF act in tandem with lower levels of cortisol in individuals when psychological distress is suspected.

BDNF circulates systemically and supports the notion that BDNF may play an important role in cardiometabolic morbidity.^10^,^13^ Therefore, we aimed to investigate associations between cardiometabolic risk markers (glycated haemoglobin, blood pressure and silent ischaemic events), cortisol and cortisol:BDNF ratio in a bi-ethnic cohort.

## Methods

This sub-study is part of the Sympathetic Activity and ambulatory Blood Pressure in Africans (SABPA) study carried out in 2008–2009 and described elsewehere.^14^ The population consisted of 409 teachers from the Dr Kenneth Kaunda Education District, South Africa. Selection ensured a socio-economically similar population despite differences in cultural characteristics.

Exclusion criteria included tympanum temperature above 37.5°C, the use of anti-depressants, α- and β-blockers, and blood donors or individuals vaccinated within a period of three months prior to data collection. Additionally we excluded cortisone users (n = 3), and the final sample comprised 406 individuals.

Participants were fully informed with regard to the study procedure and signed an informed consent form. The study was approved by the Ethics Review Board of the North-West University (NWU-00036-07-S6).

During the 48-hour clinical data-collection process, ambulatory blood pressure (ABPM), electrocardiogram (Cardiotens CE120®, Meditech, Budapest, Hungary) and accelerometer measures were obtained (Actical®, Mini Mitter, Montreal, Quebec). The BP apparatus was fitted before 09:00, with an appropriately sized cuff on the non-dominant side of the participant. The participants were asked to record abnormalities such as nausea, feeling stressed or having a headache on a 24-hour diary card. The apparatus was pre-programmed to measure blood pressure every 30 minutes (08:00–22:00) and hourly (22:00–06:00).

The ABPM and ECG data were analysed using the CardioVisions 1.19 Personal Edition software (Meditech). An average 24-hour systolic blood pressure (SBP) of ≥ 130 mmHg and/or diastolic blood pressure (DBP) of ≥ 80 mmHg were used as the criteria to define hypertension.^16^

Silent ischaemia was assessed by two-channel ECG recordings (Cardiotens CE120®) for 20 seconds at five-minute intervals. An ischaemic event was defined according to the following criteria: horizontal or descending ST-segment depression of 1 mm, duration of ST-segment episode lasting for one minute, and a one-minute interval from the preceding episode.^15^

At 16:30, participants were transported to the North-West University’s Metabolic Unit Research Facility where they were introduced to the experimental procedures. They completed psychosocial questionnaires under supervision of a registered clinical psychologist. They received a standardised dinner and were advised to go to bed at 22:00 and to fast overnight. At 05:45 they were woken, and the devices were removed after the last ambulatory recording at 06:00. Anthropometric measurements and fasting blood samples followed.

The participant’s daily physical activity was monitored over 24 hours, considering resting metabolic rate, with the Actical® activity monitor (Mini Mitter Co, Inc, Bend, OR; Montreal, Quebec, Canada). Gamma-glutamyl transferase (γ-GT) and cotinine levels were used to assess alcohol intake and smoking habits.

Anthropometric measurements were done in triplicate by level two-accredited anthropometrists using calibrated instruments (Precision health scale, A & D Company, Tokyo, Japan; Invicta Stadiometer IP 1465, Invicta, London UK). Body mass and height of the participants were measured while remaining in underwear, for accuracy. Body surface area (BSA) (in m^2^) was calculated according to the Mosteller formula. The mean of three measurements was used to ensure accuracy. Inter- and intra-observer variability was found to be less than 10%.

Fasting blood samples were obtained from the ante-brachial vein branches with a winged infusion set using standardised protocol, and were stored at –80°C until batch assay. Sequential multiple analysers analysed serum gamma-glutamyl transferase, high-sensitivity C-reactive protein (hsCRP) (low-grade inflammation was defined when hsCRP was > 3 mg/l), cotinine and HbA_1c_ levels (glycated haemoglobin) (Konelab 20i, Thermo Scientific, Vantaa, Finland; Unicel DXC 800- Beckman and Coulter®, Germany and the Integra 400, Roche, Switzerland respectively).

Quantikine colorimetric-sandwich immunoassays from R & D Systems (catalogue number: DBD00) were used to determine serum BDNF levels with an intra-assay and inter-assay precision of 3.8–6.2% and 7.6–11.3%, respectively. Serum cortisol samples were obtained before 09:00, avoiding the cortisol awakening responses (CAR),^17^ and analysed with ECLIA on Elecsys 2010, Roche. The cortisol:BDNF ratio was calculated by converting cortisol to the same SI unit as BDNF (from nmol/ml to pg/ml) to obtain cortisol:BDNF.

## Statistical analysis

Data analysis was done using Statsoft (Statistica V.12). The Shapiro–Wilks test ascertained normality of data, and skewed data were log normalised (log physical activity, log cotinine levels, log γ-GT). Multiple comparisons were not done and a priori hypotheses for all tests were performed.

Baseline characteristics of the bi-ethnic population were compared via independent t-tests. Chi-squared (χ^2^) tests computed proportions and prevalence. The raw data are presented as mean ± standard deviation in the descriptive table (Table 1) to ensure clarity of clinical observations.

**Table 1:** Characteristics of a South African bi-ethnic gender cohort

**	*Africans*	*Caucasians*	**
*Variables*	*(n = 197)*	*(n = 209)*	*p-values*
Confounders			
Age(years)	44.4 ± 8.2	45.0 ± 10.9	0.49
Body mass index (kg/m^2^)	30.1 ± 7.0	27.6 ± 5.9	< 0.001
Body surface area (m^2^)	1.9 ± 0.2	2.0 ± 0.3	< 0.001
Physical activity (kcal/day)	2670 ± 794.4	3112 ± 1596.5	< 0.001
Cotinine (ng/ml)	27.5 ± 61.3	22.71 ± 77.5	0.5
γ-Glutamyl transferase (U/l)	66.3 ± 83.0	26.91 ± 33.9	< 0.001
Potential cardiometabolic risk markers			
Cortisol (nmol/l)	358.03 ± 151.63	384.11 ± 159.9	0.093
BDNF (pg/ml)	1411.6 ± 652.3	1687.3 ± 888.1	< 0.001
Cortisol:BDNF ratio	126.6 ± 114.4	102.7 ± 71.6	0.012
C-reactive protein (mg/l)	8.55 ± 10.56	3.1 ± 3.88	< 0.001
Cholesterol (mmol/l)	4.6 ± 1.16	5.5 ± 1.28	< 0.001
HbA_1c_ (%)	6.1 ± 1.2	5.5 ± 0.42	< 0.001
24-h SBP (mmHg)	133 ± 16	124 ± 12	< 0.001
24-h DBP (mmHg)	83 ± 11	77 ± 8	< 0.001
12-lead ECG HR (bpm)	68 ± 13	66 ± 11	0.003
Hypertension, n (%)	43 (26.54)	18 (9.33)	< 0.001
Medications				
Hypertensive treatment, n (%)	69 (35.03)	27 (12.92)	< 0.001
Statins, n (%)	2 (1.23)	9 (4.67)	0.05
Diabetes medication, n (%)	10 (5.08)	2 (0.96)	0.01
CRP > 3 mg/l, n (%)	106 (65.35)	39 (20.97)	< 0.001
HIV status, n (%)	19 (9.5)	0	< 0.001

Values presented as arithmetic mean ± SD.

BDNF, brain-derived neurotrophic factor; HbA1c, glycated haemoglobin; 24-h hypertension (SBP ≥ 130 mmHg and/or DBP ≥ 80 mmHg); HR, heart rate;

CRP, C-reactive protein; physical activity, 24-h total energy expenditure, considering resting metabolic rate; n, prevalence (%).

General linear models determined interactions on the main effects (ethnic × gender) for all cardiometabolic variables independent of a priori confounders (age, body surface area, log physical activity, log cotinine and log γ-GT). There after ANCOVAs, using least-square means, compared bi-ethnic gender groups while adjusting for a priori confounders.

Pearson and partial correlation analyses determined unadjusted and adjusted associations between HbA_1c_ level, 24-hour BP, silent ischaemia and cortisol level, as well as cortsol:BDNF, independent of a priori covariates. Forward stepwise linear regression analyses determined associations in several models between dependent variables: HbA_1c_ level, blood pressure, ischaemia and the independent variables: cortisol, cortisol:BDNF and a priori covariates in the separate ethnic gender groups.

Sensitivity analyses: forward stepwise linear regression analyses were repeated after excluding HIV-positive status teachers, and hypertension and diabetes medication users. Significant values were noted as p ≤ 0.05.

## Results

In Table 1, the black African group portrayed a more vulnerable cardiometabolic profile than the Caucasians. They consumed more alcohol, had higher BP and mean pre-diabetes (HbA_1c_) levels, lower BDNF levels (p < 0.001), larger cortisol:BDNF ratios (p = 0.012) and a higher mean 24-hour hypertensive state. Caucasians were more physically active compared to the Africans. Both ethnic groups’ cortisol levels were within the normal range of 138–635 nmol/l but the Africans’ cortisol levels showed a tendency towards lower levels (p = 0.093) than their Caucasian counterparts.

In Table 2, ANCOVA analyses, considering a priori covariates, showed that the cortisol level was lower in African men, while BDNF was lower in African women compared to their Caucasian counterparts. The African gender groups showed increased hyperglycaemia, low-grade inflammation, 24-hour BP values and heart rate compared to their Caucasian counterparts.

**Table 2:** Comparing differences in cardiometabolic risk markers in ethnic male and female groups

*Risk markers*	*African men (n = 99)*	*Caucasian men (n = 101)*	*African women (n = 98)*	**Caucasian women (n = 108)
Unadjusted cardiometabolic risk markers				
γ-Glutamyl transferase (U/l)	84.83 (70.1–99.5)	34.83 (20.2–49.4)**	46.9 (35.9–57.9)	19.9 (9.6–30.2)**
Cholesterol (mmol/l)	4.64 (4.4–4.9)	5.63 (5.4–5.9)**	4.4 (4.1–4.7)	5.57 (5.3–5.8)**
C-reactive protein,(mg/l)	5.93 (4.6–7.2)	1.71 (0.4–3.0)**	11.14 (9.4–12.8)	4.41 (2.8–6)**
HbA_1c_ (%)	6.29 (6.1–6.5)	5.60 (5.4–5.8)**	5.85 (5.7–6.1)	5.40 (5.2–5.6)**
Adjusted cardiometabolic risk markers				
24-h SBP (mmHg)	140 (137–142)	125 (123–128)**	128 (126–131)	121 (119–123)**
24-h DBP (mmHg)	89 (87–91)	78 (77–80)**	79 (77–80)	74 (72.7–76)**
24-h heart rate (bpm)	79 (77–82)	72 (70–74)**	80 (78–82)	76 (73.7–77)**
Silent ischaemic events, score	10.3 (6.9–13.6)	1.1 (0.02–4.4)**	2.8 (1.7–4.0)	3 (1.9–4.0)
Cortisol (nmol/l)	364.38 (334.59–394.2)	410.41 (380.86–440.0)*	343.95 (308.5–379.4)	368.87 (335.7–402.1)
BDNF (pg/ml)	1250.41 (1095.8–1405.1)	1426.28 (1272.9–1579.7)	1599.62 (1429–1770.3)	1925.77 (1765.3–2086.3)**
Cortisol:BDNF ratio	142.8 (118.2–167.4)	139.67 (115.3–164.1)	96.16 (82.9–109.4)	80.43 (67.9–92.9)

Values depicted as mean (± 95% confidence interval) and proportions as n (%). Adjustments were made for age, body surface area, log physical activity, log cotinine and log γ-GT. HbA1c, glycated haemoglobin; BDNF, brain-derived neurotrophic factor; DBP, diastolic blood pressure; SBP, systolic blood pressure.

*p ≤ 0.05; **p ≤ 0.01.

Pearson correlations showed inverse associations between silent ischaemia and cortisol:BDNF (r = 0.34; p = 0.001) in the African male cohort but not in any of the other ethnic gender groups (data not shown). When considering a priori covariates (Tables 3, 4), forward stepwise linear regressions confirmed similar trends with a stronger association between silent ischaemia [Adj R2 0.22; β 0.40 (0.2–0.6); p < 0.01] and cortisol:BDNF ratio in African men, but not in any of the other ethnic gender groups.

**Table 3:** Independent associations between cardiometabolic risk markers, cortisol as well as cortisol:BDNF in a South African cohort

**	*South African cohort (n = 406)*			
**	*HbA_1c_*		*Silent ischaemia*	
**	*β (95% CI)*	*p-value*	*β (95% CI)*	*p-value*
Adjusted R^2^	0.16		0.10	
Cortisol	0.1 (0.0–0.2)	0.03	0.26 (0.2–0.4)	< 0.01
Ethnicity	–0.34 (–0.4– –0.2)	< 0.01	–0.13 (–0.2–0.0)	< 0.01
Gender	–0.09 (–0.2–0.0)	0.08	–	
Age	0.15 (0.1–0.2)	< 0.01	0.11 (0.0–0.2)	0.02
Body surface area	0.17 (0.1–0.3)	< 0.01	–	

Adjusted R2	0.15		0.10	
Cortisol:BDNF	-		0.26 (0.2–0.4)	< 0.01
Ethnicity	–		–0.13 (–0.2–0.0)	< 0.01
Age	–		0.11 (0.0–0.2)	0.02

HbA_1c_, glycated haemoglobin. Additional covariates included: log physical activity, log cotinine levels, log gamma glutamyl transferase. Where ethnicity (1 = African, 2 = Caucasian); gender (1 = men, 2 = women).

**Table 4:** Independent associations between cardiometabolic risk markers, cortisol as well as cortisol:brain derived neurotrophic factor (BDNF) in an African male cohort

**	*HbA1c*		*24-h SBP*		*24-h DBP*		*Silent ischaemia*	
**	*β (95% CI)*	*p-value*	*β (95% CI)*	*p-value*	*β (95% CI)*	*p-value*	*β (95% CI)*	*p-value*
Adjusted R^2^	0.16		0.29		0.21		0.12	
Cortisol	0.21 (0.0–0.4)	0.04	0.23 (0.1–0.4)	0.01	0.23 (0.1–0.4)	0.01	0.01 0.18 (0.0–0.4)	0.07
GGT	-		0.14 (0.0–0.3)	0.10	0.22 (0.0–0.4)	0.02	-	
Age	-		0.30 (0.1–0.5)	< 0.01	0.20 (0.0–0.4)	0.04	0.33 (0.1–0.5)	< 0.01
Log physical activity	–		0.30 (0.1–0.5)	0.02	0.28 (0.0–0.5)	0.08	–	
Body surface area	-		0.18 (–0.1–0.4)	0.14	0.15 (–0.1–0.4)	0.26	–	

Adjusted R^2^		< 0.10		0.26		0.15		0.22
Cortisol:BDNF	0.20 (0.0–0.4)	0.03	–		–		0.40 (0.2–0.6)	< 0.01
Log cGGT	–		–		–		–	
Age	0.32 (0.1–0.5)	< 0.01	–		–		0.36 (0.2–0.5)	< 0.01
Log physical activity	0.29 (0.0–0.5)	0.02		–	–		0.16 (0.0–0.4)	0.09
Body surface area	0.21 (0.0–0.5)	0.09		–	–		–	

HbA_1c_, glycated haemoglobin; SBP, systolic blood pressure; DBP, diastolic blood pressure; log cGGT, log gamma-glutamyl transferase.

Cortisol level was positively associated with HbA_1c_ level, and 24-hour BP with a tendency for silent ischaemia (p = 0.07) in the African men only. No change in the outcome was demonstrated after adjustment for HIV-positive status, hypertension and diabetes medication use.

## Discussion

Our objectives were to investigate associations between cortisol, the cortisol:BDNF ratio and cardiometabolic risk markers, specifically HbA_1c_ level, ambulatory BP and silent ischaemia in a bi-ethnic gender cohort. Overall, African men showed a poorer cardiometabolic profile accompanied by lower cortisol levels when compared to their Caucasian counterparts. Attenuated cortisol levels therefore seem to act as the driving force in the cortisol:BDNF ratio and may ultimately down-regulate BDNF.

The novel ratio of cortisol:BDNF may sustain cardiometabolic risk and induce neurodegeneration. Cardiometabolic morbidity further increased in the African men as their reduced coronary perfusion, as evidenced in the number of 24-hour silent ischaemic events, would increase BP as a compensatory mechanism to maintain homeostasis.

Despite the fact that a pre-diabetic state was demonstrated in the African gender cohort, it was not directly associated with cortisol:BDNF ratio. In another SABPA sub-study, chronic hyperglycaemia was shown to facilitate endothelial dysfunction and susceptibility to stroke risk in the African male cohort.^17^ Indeed, Hamer et al.^18^ also demonstrated associations between glucose homeostasis as assessed by HbA_1c_ concentration and coronary artery calcification. A hyperglycaemic state therefore predisposes to cardiometabolic morbidity in the presence of emotional distress markers such as cortisol:BDNF.

A profile of blunted cortisol and norepinephrine metabolite responses were associated with structural wall remodelling in a depressed SABPA African male cohort.^12^ The attenuated cortisol levels of the current African sub-group may therefore support the notion of increased chronic distress.^19^,^20^

Attenuated BDNF levels in Africans might also induce decreased neuroplasticity, vulnerability to depression and cardiovascular risk^6^ In this study, cortisol:BDNF ratio was associated with silent ischaemia, suggesting possible downregulation of BDNF, and implicating a central neural regulatory role.^10^ In animal studies, high concentrations of BDNF in hypothalamic nuclei and neurons secreting CRF support the role of BDNF in the stress response.^6^ During chronic stress, a maladaptive cortisol response is elicited due to structural changes in the HPA axis.^21^-^23^ A state of psychological distress in Africans may therefore be present.^12^,^19^,^20^

Indeed, we have confirmed that chronic depression in the SABPA African teachers’ cohort was associated with microvascular dysregulation and perfusion deficits.^21^ If this state is further supported by high levels of alcohol abuse, the central depressant effect of alcohol may disturb central cardiometabolic homeostasis.^21^ Chronic psychological stress induces sympathetic hyperactivity,^17^ higher circulating levels of catecholamine and cortisol, and ultimately down-regulation will occur.^12^,^22^-^24^ Therefore, the lower cortisol level may act as the driving force behind a possible down-regulated BDNF.

The cortisol:BDNF ratio in African men suggests a susceptibility to increased cardiometabolic risk. Indeed, cortisol:BDNF is associated with silent ischaemia in African men and impairs central autoregulation, explaining the compensatory increases in blood pressure to maintain homeostasis.^3^,^14^,^19^

During chronic stress, sympathetic hyperactivity and increased norepinephrine and cortisol levels will elicit vasoconstrictive responses and reduce perfusion in the coronary circulation.^12^,^23^ Furthermore, susceptibility to emotional distress as well as HPA-axis disturbance is enhanced when using defensive coping mechanisms.^14^ Defensive coping facilitated autonomic dysfunction or sympathetic hyperactivity in the current African male cohort.^14^ This underpins the importance of a central regulatory control system where higher emotional demands impact on cardiometabolic health.

Cortisol can further induce or exacerbate down-regulation of BDNF during chronic stress conditions.^6^,^10^ Lower levels of BDNF have been associated with depression,^6^ and recently cardiometabolic risk.^10^ Down-regulation and the aftermath of low BDNF levels impair neuroplasticity and homeostasis.^11^,^13^,^23^,^24^ With BDNF down-regulation, dysregulation of the HPA axis may also occur, as both BDNF and cortisol critically impact on the stress response.^24^ This suggests that central neural control dysregulation enforces a disturbed cortisol:BDNF ratio, augmenting silent ischaemia and overburdening the heart. This is the first time this ratio has been reported, and further research is needed to support the findings and significance.

Susceptibility to emotional distress may be an underlying factor in the observed differences in the bi-ethnic gender cohort. We suggest that attenuated cortisol levels may act as the driving force to downregulate BDNF, increasing cardiometabolic risk and reducing coronary perfusion during psychological distress.

One limitation of the study is that a very specific population was studied and results may vary across populations, depending on similar lifestyle and behavioural factors. Another limitation is the cross-sectional nature of the study where causality cannot be inferred. ECG assessment of silent ischaemia showed a sensitivity of 68% and a specificity of 77%,25 therefore further studies are needed to support silent ischaemic events, such as troponin T determinations, in order to confirm reduced blood supply to the heart. We recommend prospective analyses to underpin downregulation of the cortisol:BDNF pathway, as well as the use of a validated depression score to substantiate chronic emotional distress and its relationship with cortisol:BDNF ratio.

## Conclusion

Central neural dysregulation may mediate cortisol levels as the driving force in the cortisol:BDNF ratio, as it seemingly disturbs BDNF levels during chronic stress. A possible down-regulation may lead to neurodegeneration and add to cardiometabolic risk in Africans. The combined impact of cortisol and BDNF levels associated with silent ischaemia may increase future coronary artery disease risk via compensatory increases in blood pressure.
